# The impact of physical activity on healthy ageing trajectories: evidence from eight cohort studies

**DOI:** 10.1186/s12966-020-00995-8

**Published:** 2020-07-16

**Authors:** Darío Moreno-Agostino, Christina Daskalopoulou, Yu-Tzu Wu, Artemis Koukounari, Josep Maria Haro, Stefanos Tyrovolas, Demosthenes B. Panagiotakos, Martin Prince, A. Matthew Prina

**Affiliations:** 1 Department of Health Service and Population Research, King’s College London, Institute of Psychiatry, Psychology and Neuroscience, David Goldberg Centre, De Crespigny Park, London, SE5 8AF UK; 2 Department of Infectious Disease Epidemiology, London School of Hygiene & Tropical Medicine, Faculty of Epidemiology and Population Health, London, UK; 3 Parc Sanitari Sant Joan de Déu, Universitat de Barcelona. Fundació Sant Joan de Déu, Dr Antoni Pujades, 42, 08830, Sant Boi de Llobregat, Barcelona, Spain; 4 Instituto de Salud Carlos III, Centro de Investigación Biomédica en Red de Salud Mental, CIBERSAM, Madrid, Spain; 5 Department of Nutrition and Dietetics, School of Health Science and Education, Harokopio University, Athens, Greece

**Keywords:** Growth mixture modelling, Lifestyle behaviours, Health metric, Data harmonisation, Physical activity

## Abstract

**Background:**

Research has suggested the positive impact of physical activity on health and wellbeing in older age, yet few studies have investigated the associations between physical activity and heterogeneous trajectories of healthy ageing. We aimed to identify how physical activity can influence healthy ageing trajectories using a harmonised dataset of eight ageing cohorts across the world.

**Methods:**

Based on a harmonised dataset of eight ageing cohorts in Australia, USA, Mexico, Japan, South Korea, and Europe, comprising 130,521 older adults (*M*_age_ = 62.81, *SD*_age_ = 10.06) followed-up up to 10 years (*M*_follow-up_ = 5.47, *SD*_follow-up_ = 3.22)*,* we employed growth mixture modelling to identify latent classes of people with different trajectories of healthy ageing scores, which incorporated 41 items of health and functioning. Multinomial logistic regression modelling was used to investigate the associations between physical activity and different types of trajectories adjusting for sociodemographic characteristics and other lifestyle behaviours.

**Results:**

Three latent classes of healthy ageing trajectories were identified: two with stable trajectories with high (71.4%) or low (25.2%) starting points and one with a high starting point but a fast decline over time (3.4%). Engagement in any level of physical activity was associated with decreased odds of being in the low stable (OR: 0.18; 95% CI: 0.17, 0.19) and fast decline trajectories groups (OR: 0.44; 95% CI: 0.39, 0.50) compared to the high stable trajectory group. These results were replicated with alternative physical activity operationalisations, as well as in sensitivity analyses using reduced samples.

**Conclusions:**

Our findings suggest a positive impact of physical activity on healthy ageing, attenuating declines in health and functioning. Physical activity promotion should be a key focus of healthy ageing policies to prevent disability and fast deterioration in health.

## Background

Ageing has been associated with an increased risk of non-communicable diseases, frailty [1] and disability [2]. To address economic, health and social care burdens related to these adverse health conditions, maintenance of good health in later life has become a key priority for ageing research and health policy planning. In this regard, the latest *World Report on Ageing and Health* by the World Health Organization (WHO) [3] has provided a framework for the study and promotion of healthy ageing. In this report, WHO defined healthy ageing as the “process of developing and maintaining the functional ability that will enable older people to do the things that matter to them”, and this process is not homogeneous in the population [4].

Physical activity promotion has been suggested to reduce the risk of developing non-communicable diseases [5] and the healthcare expenditure [6], and to increase the satisfaction with one’s life and with the ageing process [7]. Evidence from epidemiological studies has also shown that physical activity is strongly associated with healthier ageing trajectories [8, 9], better cognitive function trajectories [10] and improved frailty trajectories [11]. In particular, a recent meta-analysis [12] has summarised the findings from 23 longitudinal studies focusing on the associations between physical activity and healthy ageing and suggested that physically active older adults (defined by any levels of exercises or activities) had almost 40% increased odds of experiencing healthy ageing compared to their non-physically active counterparts. However, there was a high heterogeneity in the conceptualisation of healthy ageing, with different studies using different combinations of variables related to physical performance (functioning and disability), diseases, mental health, and survival to a specific age [12]. Moreover, most existing studies mainly focused on population average trajectories of healthy ageing and did not consider the existence of subgroups in the older population that may exhibit different trajectories than the majority of it [3]. This might be due to limited sample size and statistical power to detect subgroups of different trajectories within a single study population. To provide novel insights on how physical activity can influence trajectories of healthy ageing in different populations, it is necessary to incorporate data from multiple cohort studies and use comparable measures for physical activity and healthy ageing.

In this study, we aim to investigate the impact of physical activity on trajectories of healthy ageing, while taking into account sociodemographic factors and other correlated lifestyle factors, by using a harmonised dataset of eight longitudinal cohort studies across the world. Given the strength of large sample sizes, we identified various types of healthy ageing trajectories in the study population and examined their relationships with different levels of physical activity.

## Methods

### Study sample

This study was based on the Ageing Trajectories of Health: Longitudinal Opportunities and Synergies (ATHLOS) project (http://athlosproject.eu/). The aim of the ATHLOS project was to harmonise data from 17 international ageing cohort studies [13] and to investigate trajectories of healthy ageing and their potential determinants in different older populations. Data harmonisation is a technique where data from different studies and with different format are transformed and merged together in order to produce one cohesive dataset. Bringing together 17 ageing cohorts across the world, the ATHLOS harmonised dataset highlights several advantages of data harmonisation: first, it allows researchers to increase the size of the study population and hence the statistical power of the analyses; second, it enables the investigation of those trajectories and any potential determinants independently from the older adults’ settings and backgrounds while adjusting the analyses for differences in the source of the data, thus providing more generalisable evidence. The ATHLOS consortium followed the Maelstrom Research guidelines [14]. Harmonisation of variables across studies was based on an iterative process of consensus of experts, which is transparently documented and publicly available harmonisation reports (https://github.com/athlosproject/athlos-project.github.io/). Further details on the included cohorts and the harmonised variables is also available in the cohort profile of the ATHLOS project [13]. To carry out longitudinal analyses and identify different trajectories of healthy ageing, we focused on cohorts with three or more available waves. This included the Australian Longitudinal Study of Ageing (ALSA), the English Longitudinal Study of Ageing (ELSA), the Study on Cardiovascular Health, Nutrition and Frailty in Older Adults in Spain (ENRICA), the Health and Retirement Study (HRS), the Japanese Study of Ageing and Retirement (JSTAR), the Korean Longitudinal Study of Ageing (KLOSA), the Mexican Health and Aging Study (MHAS) and the Survey of Health Ageing and Retirement in Europe (SHARE). In the present study, we excluded participants with information on health only at a single time point. The analytic sample comprised 130,521 individuals from 26 countries (i.e. Australia, Austria, Belgium, Czech Republic, Denmark, Estonia, France, Germany, Greece, Hungary, Ireland, Israel, Italy, Japan, Luxembourg, Mexico, Netherlands, Poland, Portugal, Slovenia, South Korea, Spain, Sweden, Switzerland, United Kingdom, and USA), whereas the excluded sample comprised 78,279 individuals.

### Measures

#### Healthy ageing score

Based on the WHO framework healthy ageing [3, 15], the ATHLOS healthy ageing score was constructed by using a two-parameter logistic item response theory (IRT) model with 41 items related to intrinsic capacity and functional ability (see **Table****S1**, Supplementary Material). Heterogeneities in the cohort-specific datasets were analysed and addressed to obtain the common scale. The IRT model converged successfully with an excellent fit (RMSEA = 0.03, TLI = 0.99, and CFI = 0.99), and had a marginal reliability of 0.83. The estimated latent scores obtained for each participant at each time point were rescaled to a range between 0 and 100, with higher numbers indicating better health status.

#### Physical activity

Two harmonised variables were used to assess physical activity at baseline: frequency of vigorous exercise and frequency of less vigorous exercise throughout the week (in days). Both variables had five categories (never, once per week, 2/3 times/days per week, 4–5 times/days per week, and 6–7 times/days per week) that were recoded from the original variables in which participants were asked for the number/frequency of “vigor sessions” (ALSA), “vigorous physical activities” (HRS), “strenuous activity” (JSTAR), “exercise” (KLOSA), or “sports or activities that are vigorous” (SHARE), regarding vigorous exercise; or “less vigor sessions” (ALSA), “light physical activities” (HRS), “light exercise” (JSTAR), or “activities requiring a moderate level of energy” (SHARE), regarding less vigorous exercise. The last two categories of the harmonised variables were grouped into 4+ times/days per week in this study. As not all included studies had this information, we created an aggregated variable of physical activity (yes/no) to minimise missingness. To create the latter variable, we considered the following harmonised questions (the corresponding response options appear between parentheses): level of physical activity (high, fair, low, not at all), frequency of vigorous and less vigorous exercise (never, once per week, 2/3 times/days per week, 4+ times/days per week), engagement in vigorous exercise during the last 2 weeks (yes, no), frequency of vigorous exercise activities in the last 2 weeks (number), and time spent doing vigorous exercise in the last 2 weeks (in minutes). Participants were categorised as physically active if at least one of the following criteria was met: had “high” or “fair” level of physical activity; engaged in less vigorous or more vigorous exercise 2+ times per week or more; engaged in vigorous exercise (either with a “yes” to the frequency question, or with an answer different from zero to the question on the time spent doing vigorous exercise), or had 5 or more time vigorously exercised in the last 2 weeks. The presence of these different questions by study is shown in **Table****S2** (Supplementary Material).

#### Covariates

Covariates included age, gender, study, education, wealth, smoking and alcohol consumption. Education was categorised into three groups (primary or less, secondary and tertiary education) based on qualification. Wealth was measured using income and financial information and divided into quintiles (1st: least) within each cohort. As in the case of physical activity, we harmonised measures of alcohol consumption and smoking that allowed the least amount missing data across studies. Thus, we considered if participants reported ever having smoked or consumed alcohol either at baseline or any of the follow-up waves.

### Statistical analyses

To investigate the heterogeneity in the longitudinal trajectories of health, we employed the framework of growth mixture modelling (GMM) [16], which allows identifying unobserved groups (latent classes) of individuals who exhibit different patterns of health change over time. Since most cohorts had an investigation period up to 10 years and carried out follow-up waves every 2 years, here we focused on the first 10 years of follow-up and built trajectories by biennial intervals (year 0, 2, 4, 6, 8 and 10). Hence, mean elapsed time across waves was 2 years (variance is not available).

Following Ram and Grimm indications [16], we performed a single-class model (i.e. latent growth curve model) to find the best representation of change in the overall sample (linear or quadratic change). We used the best fitting model [lower Bayesian Information Criterion (BIC)] as baseline model against which we compared subsequent models with different number of latent classes, ranging from two to five. We computed these models considering either linear or quadratic change. To decide on the final number of latent classes (i.e. trajectories), we employed the Bayesian Information Criterion (BIC), the sample-size adjusted BIC (SABIC), entropy values, the Lo-Mendel-Rubin likelihood ratio test (LMR-LRT), and the sample size of the smallest class (no less than 1% of the total sample in any class) [17, 18]. The entropy of the model was prioritised as selection criteria in order to optimise the separation between the classes [16]. The models were estimated using maximum likelihood with errors robust to non-normality and non-independence of observations (MLR) [19], and missing data were assumed to be missing at random (MAR). Moreover, we used 500 random sets of starting values for the parameters, along with 250 final optimisations, to prevent the models from converging on a local solution. In order to balance the flexibility of the models with the estimation ability, residual variances and covariances of the growth parameters were estimated but constrained to be equal across latent classes to avoid estimation problems. Alternative specifications of the within-class heterogeneity such as Latent Class Growth Analysis, in which within-class heterogeneity is constrained to be zero, were not considered due to their inability to reflect the expected individual heterogeneity in the healthy ageing process [3, 4].

Once the model was selected, we used a three-step approach to estimate the association of the three physical activity variables (i.e. aggregated physical activity, frequency of vigorous exercise, and frequency of less vigorous exercise) with each latent class. In this approach, 1) the latent class model is estimated a priori without accounting for the predictor variables; 2) observations are then assigned to the most likely class using the latent class posterior distribution obtained in step 1; and 3) a new model is then estimated in order to assess the impact of the predictor variables on the class membership, fixing the measurement error to the values obtained in step 2 [20, 21]. This procedure accounts for misclassification in the second step, and the error assignments based on the highest posterior probabilities are reduced. All multinomial logistic regression models were adjusted for other lifestyle behaviours (i.e. ever smoking and drinking), as well as for baseline age, gender, study (i.e. ALSA, ELSA, ENRICA, HRS, JSTAR, KLOSA, MHAS, and SHARE), within-country household wealth quintile (1st and 2nd quintiles vs the rest), and education level (i.e. primary or less than primary, secondary, and tertiary). In these models, participants with missing data in any of the covariates were excluded from the analyses.

Since some studies did not have information on the frequency of vigorous (i.e. ELSA, ENRICA, and MHAS) or less vigorous exercise (i.e. ELSA, ENRICA, KLOSA, and MHAS), we performed a set of sensitivity analyses to investigate the robustness of our findings. In these sensitivity analyses, we performed the selected GMM model in two reduced samples (sensitivity analysis A: studies with information on vigorous exercise; sensitivity analysis B: studies with information on less vigorous exercise) and checked whether the results regarding the trajectories found and their relationship with physical activity were replicated. The Guidelines for Reporting on Latent Trajectory Studies [22] were used, and the checklist is included in the **Table****S3** (Supplementary Material). Data management and descriptive analyses were performed using Stata SE 14.2, whereas GMM models were performed in Mplus 8.1. The code is available from the corresponding author upon request.

## Results

### Trajectories of healthy ageing

A total of 130,521 participants had information in at least two measurement points. The average number of observations was 3.19 (*SD* = 1.39), and the mean duration of the follow-up was 5.47 years (*SD* = 3.22). Table 1 summarises characteristics of the analytic and excluded samples at baseline. Although some differences across included and excluded samples achieved statistically significance (to be expected due to the large sample size), the only meaningful effect size was found in the study variable, where SHARE had a higher proportion of excluded cases due to single observations from the refresh cohorts. Regarding the analytic sample, mean scores on health by years of follow-up were 70.21 (*SD* = 16.46, skewness = − 0.41, kurtosis = 2.70) for baseline, 69.02 (*SD* = 17.30, skewness = − 0.44, kurtosis = 2.77) for 2 years, 68.69 (*SD* = 17.53, skewness = − 0.42, kurtosis = 2.81) for 4 years, 67.25 (*SD* = 17.83, skewness = − 0.33, kurtosis = 2.74) for 6 years, 66.99 (*SD* = 18.45, skewness = − 0.31, kurtosis = 2.50) for 8 years, and 65.78 (*SD* = 18.43, skewness = − 0.27, kurtosis = 2.50) for 10 years.

**Table 1:** Baseline characteristics of analytic and excluded samples

		Analytic sample (*N* = 130,521)	Excluded sample (*N* = 78,279)	*t* / χ^2^	Effect size
Age, M (SD)		62.80 (10.06)	62.31 (11.68)	10.29***	0.05
Gender, N (%)	Female	72,312 (55.40)	40,632 (51.91)	0.32	0.001
	Male	58,063 (44.49)	32,453 (41.46)		
	*Missing*	146 (0.11)	5194 (6.64)	–	–
Wealth, N (%)	1st quintile	26,406 (20.23)	13,702 (17.50)	266.04***	0.04
	2nd quintile	25,419 (19.48)	13,064 (16.69)		
	3rd quintile	24,760 (18.97)	13,663 (17.45)		
	4th quintile	24,040 (18.42)	14,162 (18.09)		
	5th quintile	23,657 (18.13)	14,892 (19.02)		
	*Missing*	6239 (4.78)	8796 (11.24)	–	–
Education, N (%)	Less than primary/primary	43,469 (33.3)	22,181 (28.34)	488.55***	0.05
	Secondary	62,821 (48.13)	38,425 (49.09)		
	Tertiary	21,834 (16.73)	14,666 (18.74)		
	*Missing*	2397 (1.84)	3007 (3.84)	–	–
Study, N (%)	ALSA	1843 (1.41)	244 (0.31)	27,000***	0.36
	ELSA	14,483 (11.1)	4006 (5.12)		
	ENRICA	2513 (1.93)	6 (0.01)		
	HRS	32,934 (25.23)	4382 (5.60)		
	JSTAR	3695 (2.83)	3573 (4.56)		
	KLOSA	8928 (6.84)	1326 (1.69)		
	MHAS	12,647 (9.69)	8719 (11.14)		
	SHARE	53,478 (40.97)	56,023 (71.57)		
Health score, M (SD)		70.21 (16.46)	70.38 (17.81)	−2.05*	−0.0004
Physical activity (aggregated), N (%)	No	49,622 (38.02)	21,999 (28.10)	1300***	0.08
	Yes	80,001 (61.29)	50,555 (64.58)		
	*Missing*	1397 (0.69)	5725 (7.31)	–	–
Frequency of vigorous physical activity, N (%)	Never	42,244 (32.37)	26,326 (33.63)	3300***	0.15
	Once per week	20,738 (15.89)	12,865 (16.43)		
	2/3 per week	7129 (5.46)	882 (1.13)		
	4+ per week	23,789 (18.23)	19,278 (24.63)		
	*Missing*	36,621 (28.06)	18,928 (24.18)	–	–
Frequency of less vigorous physical activity, N (%)	Never	11,751 (9.00)	8371 (10.69)	5100***	0.19
	Once per week	16,840 (12.90)	11,984 (15.31)		
	2/3 per week	10,311 (7.90)	1145 (1.46)		
	4+ per week	44,556 (34.14)	36,324 (46.40)		
	*Missing*	47,063 (36.06)	20,455 (26.13)	–	–
Ever smoker*, N (%)	No	62,168 (47.63)	40,439 (51.66)	487.34***	0.05
	Yes	66,817 (51.19)	35,508 (45.36)		
	*Missing*	1536 (1.18)	2332 (2.98)	–	–
Ever drinker*, N (%)	No	39,019 (29.89)	29,797 (38.07)	1800***	0.09
	Yes	91,023 (69.74)	46,260 (59.10)		
	*Missing*	479 (0.37)	2222 (2.84)	–	–

*Note*. All variables correspond to participants’ baseline, except for ever drinker and ever smoker, which correspond to the whole period of participation. Effect size estimates for age and health score correspond to Cohen’s *d*; the remaining effect size estimates correspond to Cramer’s *V*, and have been computed with non-missing data only. M: mean, SD: standard deviation, N: number of cases, *t*: Student’s *t* test; χ^2^: chi-square test. *: *p* < .05; ***: *p* < .001

Table 2 provides the BIC, SABIC, adjusted LMR-LRT results, and entropy values for the one to five latent classes models. Models allowing for quadratic change showed better fit than those only allowing for linear change. The three-class solution with quadratic growth term showed the highest entropy (0.72). The trajectories resulting from all these models are depicted in **Figures****S1****,****S2****,****S3****,****S4****,****S5****,****S6****,****S7****,****S8****,****S9** (Supplementary Material). Systematically, the models with three or more classes showed the existence of a subgroup comprising around 3–4% of the population displaying a high starting point and a fast decline over time. Increasing the number of classes beyond three resulted on spliting the remaining classes, displaying a relatively stable trajectory, into additional subgroups with different starting points but parallel trajectories. The three-class model with quadratic growth was selected as the optimal solution due to its higher entropy.

**Table 2:** Fit statistics of the Latent Growth Curve Model and Growth Mixture Models

	Linear growth - Number of latent classes				
	1 class	2 classes	3 classes	4 classes	5 classes
Loglikelihood (N of parameters)	−1,661,740 (6)	−1,657,724 (9)	−1,656,300 (12)	−1,655,481 (15)	−1,654,823 (18)
BLRT		8032.7***	2846.4***	1639.3***	1314.5***
Adj. LMR-LRT		7811.6***	2768.1***	1594.2***	1278.3***
BIC	3,323,552	3,315,554	3,312,743	3,311,139	3,309,860
SABIC	3,323,532	3,315,526	3,312,705	3,311,091	3,309,803
Entropy		0.644	0.692	0.661	0.690
Group size (%)					
Class 1	100	73.5	4.1	54.1	17.2
Class 2		26.5	25.4	9.9	2.1
Class 3			70.5	3.6	43.1
Class 4				32.4	34.1
Class 5					3.5
	Quadratic growth - Number of latent classes				
	1 class	2 classes	3 classes	4 classes	5 classes
Loglikelihood (N of parameters)	−1,660,737 (10)	−1,656,703 (14)	−1,655,111 (18)	−1,654,139 (22)	
BLRT		8067.8***	3184.6***	1943.9***	
Adj. LMR-LRT		7900.1***	3118.4***	1903.5***	
BIC	3,321,592	3,313,571	3,310,434	3,308,537	
SABIC	3,321,560	3,313,527	3,310,377	3,308,467	
Entropy		0.644	0.722	0.668	
Group size (%)					
Class 1	100	73.5	25.2	34.2	
Class 2		26.5	3.4	12.2	
Class 3			71.4	3.3	
Class 4				50.3	
Class 5					

*Note.* *** *p* < .001. The model with quadratic growth and 5 classes provided an improper solution and therefore is not displayed in the table. *BLRT* Bootstrap likelihood ratio test; *Adj. LMR-LRT* Adjusted Lo-Mendell-Rubin likelihood ratio test; *BIC* Bayesian Information Criterion; *SABIC* Sample-size-Adjusted Bayesian Information Criterion

**Fig. 1:**
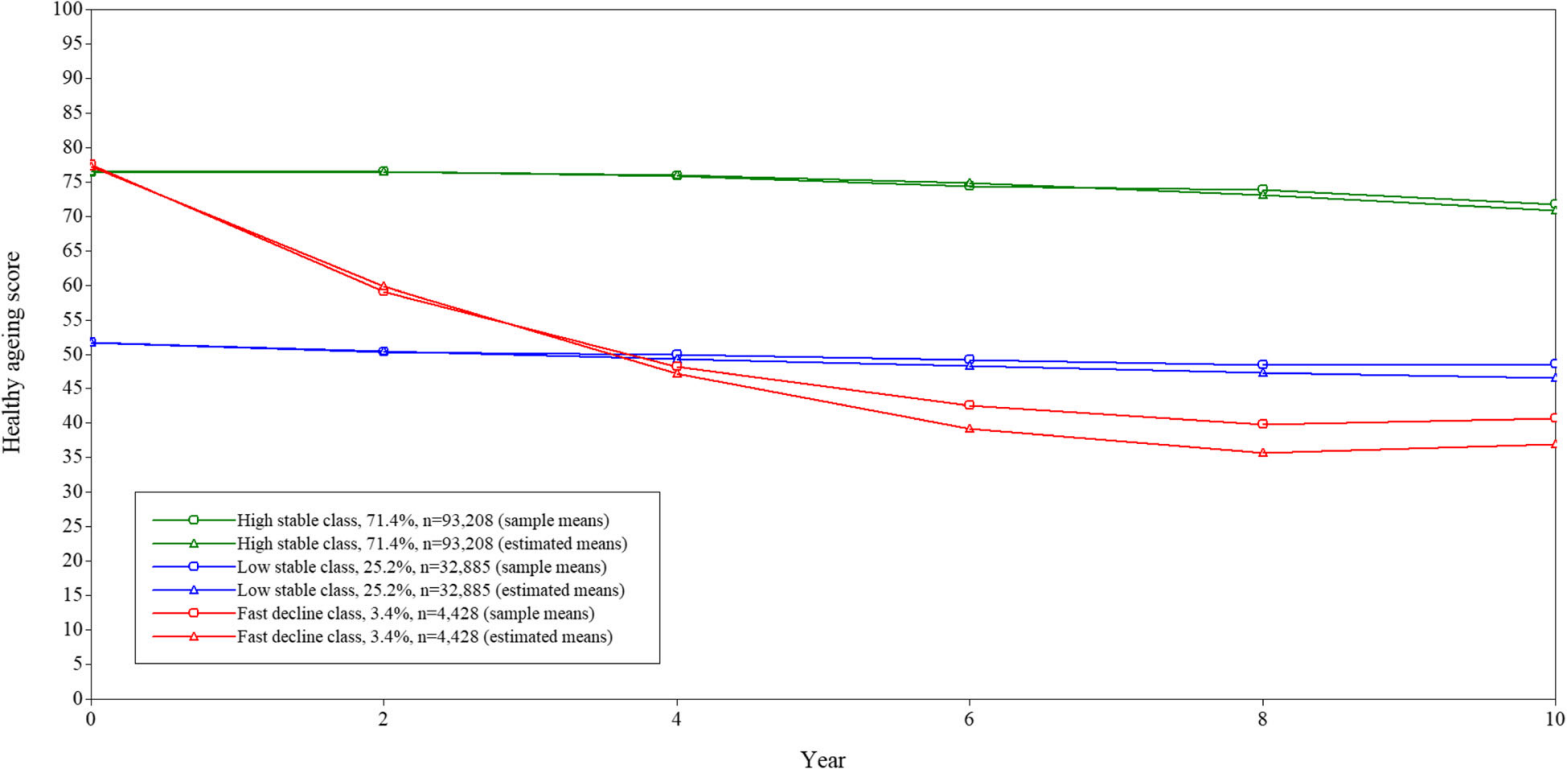
Trajectories of health according to the three latent classes Growth Mixture Model

The trajectories resulting from the selected model are depicted in Fig. 1. Latent class groups were named according to their trajectory patterns. Latent class 3 comprised the majority of the population (green line, 71.4%), displaying a high stable level of health throughout the whole period (*high stable*). Participants in latent class 1 (blue line, 25.2%) displayed a low level of health in the beginning and almost no change across the follow-ups (*low stable*). Participants in latent class 2 (red line, 3.4%) showed a baseline level of health similar to the high stable class, but a severe deterioration over time (*fast decline*). The specific estimates for the model and each of the classes are displayed in Table 3 (upper section).

**Table 3:** Estimates for the three latent classes Growth Mixture Model of healthy ageing and multinomial logistic regressions coefficients

**Latent classes (*n* = 130,521)**			
	**High stable**	**Low stable**	**Fast decline**
n (%)	93,208 (71.4)	32,885 (25.2)	4428 (3.4)
Mean Intercept (SE)	76.47 (0.08)	51.72 (0.12)	77.18 (0.31)
Mean Linear Slope (SE)	0.15 (0.03)	−0.64 (0.05)	−9.81 (0.57)
Mean Quadratic Slope (SE)	−0.07 (0.003)	0.01 (0.01)	0.58 (0.06)
Variance Intercept (SE)	83.02 (1.04)		
Variance Linear Term (SE)	2.78 (0.17)		
Variance Quadratic Term (SE)	0.03 (0.002)		
Covariance Intercept-Linear Term (SE)	−0.58 (0.30)		
Covariance Intercept-Quadratic Term (SE)	−0.01 (0.03)		
Covariance Linear-Quadratic Terms (SE)	−0.21 (0.02)		
**Physically active (*n*** **= 120,712)**			
No	Reference class	Ref.	
Yes, OR (95% CI)		0.18 [0.17, 0.19]	0.44 [0.39, 0.50]
**Vigorous physical activity (*n*** **= 90,451)**			
Never	Reference class	Ref.	
Once per week, OR (95% CI)		0.22 [0.20, 0.24]	0.43 [0.34, 0.56]
2/3 times per week, OR (95% CI)		0.18 [0.16, 0.21]	0.25 [0.16, 0.39]
4+ times per week, OR (95% CI)		0.11 [0.10, 0.12]	0.65 [0.53, 0.80]
**Less vigorous physical activity (*n*** **= 80,707)**			
Never	Reference class	Ref.	
Once per week, OR (95% CI)		0.17 [0.15, 0.19]	0.26 [0.20, 0.34]
2/3 times per week, OR (95% CI)		0.07 [0.05, 0.12]	0.11 [0.07, 0.16]
4+ times per week, OR (95% CI)		0.07 [0.06, 0.07]	0.23 [0.18, 0.28]

*Note. CI* Confidence interval; *OR* Odds ratio; *SE* Standard error. All multinomial logistic regression models are adjusted for age, gender, study, wealth, education, smoking, and alcohol drinking

### Impact of physical activity on the latent classes

Table 3 (lower section) shows the odds ratios from the multinomial logistic regression models of the latent classes on physical activity adjusted for age, gender, study, wealth, education level, ever smoking, and ever drinking. Participants engaging in vigorous or less vigorous exercise throughout the week, compared with physically inactive participants, had significantly reduced likelihood of being in the *low stable* and *fast decline* latent-classes than in the *high stable* latent-class. Similar findings were also observed for the aggregated variable of physical activity. For instance, participants that were characterised as physically inactive had 5.56 times the odds (*OR*_active_ = 0.18, 95% *CI* [0.17, 0.19]) of being in the *low stable* latent class compared to the *high stable* latent class; and 2.27 times the odds (*OR*_active_ = 0.44, 95% *CI* [0.39, 0.50]) of being in the *fast decline* latent class.

Table 4 and Table 5 display the results of the sensitivity analyses performed with the reduced samples [i.e. with the studies that had information on frequency of vigorous (A) and less vigorous exercise (B), respectively]. These analyses showed similar results regarding the number and the pattern of the health trajectories and their associations with physical activity.

**Table 4:** Estimates for the sensitivity analysis A: three latent classes Growth Mixture Model of healthy ageing and multinomial logistic regressions coefficients with reduced sample (studies with information on vigorous activity)

**Latent classes (*n* = 100,878)**			
	**High stable**	**Low stable**	**Fast decline**
n (%)	73,112 (72.5)	24,303 (24.1)	3463 (3.4)
Mean Intercept (SE)	77.06 (0.09)	51.88 (0.15)	78.67 (0.36)
Mean Linear Slope (SE)	0.17 (0.03)	−0.82 (0.05)	−9.16 (0.67)
Mean Quadratic Slope (SE)	−0.07 (0.003)	0.03 (0.005)	0.49 (0.07)
Variance Intercept (SE)	83.79 (1.15)		
Variance Linear Term (SE)	2.80 (0.20)		
Variance Quadratic Term (SE)	0.03 (0.002)		
Covariance Intercept-Linear Term (SE)	−0.07 (0.32)		
Covariance Intercept-Quadratic Term (SE)	−0.06 (0.03)		
Covariance Linear-Quadratic Terms (SE)	−0.22 (0.02)		
**Physically active (*n*** **= 96,701)**			
No	Reference class	Ref.	
Yes, OR (95% CI)		0.16 [0.15, 0.17]	0.40 [0.34, 0.46]
**Vigorous physical activity (*n*** **= 90,451)**			
Never	Reference class	Ref.	
Once per week, OR (95% CI)		0.22 [0.20, 0.24]	0.42 [0.32, 0.54]
2/3 times per week, OR (95% CI)		0.19 [0.16, 0.21]	0.22 [0.13, 0.35]
4+ times per week, OR (95% CI)		0.12 [0.11, 0.13]	0.65 [0.52, 0.82]
**Less vigorous physical activity (*n*** **= 80,707)**			
Never	Reference class	Ref.	
Once per week, OR (95% CI)		0.17 [0.15, 0.19]	0.23 [0.17, 0.31]
2/3 times per week, OR (95% CI)		0.08 [0.07, 0.09]	0.09 [0.06, 0.14]
4+ times per week, OR (95% CI)		0.07 [0.06, 0.07]	0.21 [0.17, 0.27]

*Note.* CI: confidence interval; OR: odds ratio; SE: standard error. All multinomial logistic regression models are adjusted for age, gender, study, wealth, education, smoking, and alcohol drinking

**Table 5:** Estimates for the sensitivity analysis B: three latent classes Growth Mixture Model of healthy ageing and multinomial logistic regressions coefficients with reduced sample (studies with information on less vigorous activity)

**Latent classes (*n* = 91,950)**			
	**High stable**	**Low stable**	**Fast decline**
n (%)	61,774 (67.2)	25,461 (27.7)	4715 (5.1)
Mean Intercept (SE)	78.21 (0.08)	52.51 (0.13)	78.92 (0.23)
Mean Linear Slope (SE)	0.42 (0.04)	−0.84 (0.05)	−7.97 (0.49)
Mean Quadratic Slope (SE)	−0.09 (0.004)	0.03 (0.005)	0.41 (0.05)
Variance Intercept (SE)	75.32 (1.15)		
Variance Linear Term (SE)	2.80 (0.23)		
Variance Quadratic Term (SE)	0.03 (0.002)		
Covariance Intercept-Linear Term (SE)	−1.07 (0.36)		
Covariance Intercept-Quadratic Term (SE)	0.04 (0.04)		
Covariance Linear-Quadratic Terms (SE)	−0.23 (0.02)		
**Physically active (*n*** **= 87,806)**			
No	Reference class	Ref.	
Yes, OR (95% CI)		0.18 [0.17, 0.20]	0.41 [0.36, 0.46]
**Vigorous physical activity (*n*** **= 81,556)**			
Never	Reference class	Ref.	
Once per week, OR (95% CI)		0.27 [0.25, 0.28]	0.43 [0.35, 0.53]
2/3 times per week, OR (95% CI)		0.21 [0.19, 0.24]	0.20 [0.14, 0.30]
4+ times per week, OR (95% CI)		0.14 [0.13, 0.15]	0.58 [0.47, 0.71]
**Less vigorous physical activity (*n*** **= 80,707)**			
Never	Reference class	Ref.	
Once per week, OR (95% CI)		0.21 [0.19, 0.23]	0.32 [0.25, 0.40]
2/3 times per week, OR (95% CI)		0.10 [0.09, 0.11]	0.15 [0.11, 0.20]
4+ times per week, OR (95% CI)		0.09 [0.08, 0.10]	0.24 [0.20, 0.29]

*Note. CI* Confidence interval; *OR* Odds ratio; *SE* Standard error. All multinomial logistic regression models are adjusted for age, gender, study, wealth, education, smoking, and alcohol drinking

## Discussion

### Main findings

Using a harmonised dataset of eight ageing cohorts across the world, we identified three types of healthy ageing trajectories (high stable, low stable and fast decline) and investigated how physical activity in older age was associated with these different types of trajectories. Our study suggests a positive impact of physical activity on supporting healthy ageing. Engagement in any levels of physical activity was associated with decreased odds of being in low stable or fast decline groups of healthy ageing trajectories.

### Strengths and limitations

Our study has several methodological strengths in comparison with previous research. The ATHLOS harmonised dataset covered participants from different backgrounds and the longitudinal data provided rich information on changes in health and functioning over 10 years. The large sample size and harmonised variables allowed us to explore various types of healthy ageing trajectories and their relationships with physical activity in different populations. This may increase the generalisability of the findings. Instead of focusing on presence of selected diseases or impairments, we conceptualised healthy ageing using multiple indicators of health, physical and cognitive functioning and generated a common measure across multiple cohorts.

Our findings should be considered in the light of limitations. Firstly, harmonisation as a process is retrospective and the initial studies were not designed to be harmonised. This reflects on the amount of missing data in some of the physical activity variables. To try to overcome this limitation, 1) we conducted sensitivity analyses to assess potential differences in the number and the characteristics of the latent classes due to missing values on these physical activity variables. We found that the shape and the size of the latent groups were similar across all analyses, suggesting the robustness of the results. 2) We also created the physical activity aggregated variable and employed it along with the other two physical activity variables to assess the potential impact of these different operationalisations on the associations. Even though there were some differences in the effect size estimates, the conclusions did not change based on the measure used: people that engaged in some physical activity had higher chances to be included in the group with better health. In line with this first limitation, the aggregated physical activity variable was created ad hoc in the present study to allow for the inclusion of cohort studies that used different operationalisations of physical activity. However, the sensitivity analyses performed using different categorisations of physical activity achieved similar results, thus providing evidence on the robustness of the findings involving this aggregated variable. Additionally, the information on physical activity was based on self-reported items, which may have been affected by response biases. These limitations prevent us, for instance, from providing detailed insights about the specific amount of physical activity that related to the membership to the better healthy ageing trajectory. Although we controlled for behavioural factors such as smoking and alcohol drinking, we could not control for the potential confounding effect of diet quality in our analyses [23] since that information was not available in the majority of the included studies. Moreover, the sample size in smallest latent class (i.e. *fast decline*) was relatively low, which led to wider confidence intervals. Finally, although recent research on low- and middle-income countries has found results that are consistent with the evidence presented in our study [9, 24], it is important to note that most of the countries included in this study were high-income countries. As a result of these limitations, extra caution is needed when generalising these findings.

### Interpretation of findings

The results from our study suggest that physically inactive older adults were more likely to exhibit worse trajectories of health with age than those that engaged in some form of physical activity. This corresponds to the literature and highlights the important role of being physically active in healthy ageing [12, 25]. Instead of focusing on presence of diseases or dichotomised health outcomes [25], our study shows the existence of subgroups within the older population exhibiting different trajectories of health and provides evidence on how physical activity is related to those different trajectories after adjusting for socioeconomic factors, smoking, drinking, and data source (i.e. study). It is true that variation in the healthy ageing trajectories may be the result of specific determinants (i.e. physical activity as our findings indicate) or due to the heterogeneity across countries or research settings. However, previous research in sub-studies of the ATHLOS harmonised dataset [i.e. 10/66 study [24] and MHAS study [9]] has suggested similar trajectories of healthy ageing and a similar effect of physical activity to those. Notably, the study focused on the MHAS cohort [9] adopted a similar analytical approach and identified four types of healthy ageing trajectories (high stable, moderate stable, low stable, and fast decline) in over the 10 year follow-up period and reported that physical activity was associated with lower odds of being in low stable and fast decline groups. Taking these previous research evidence and the findings of the present study altogether, it may be assumed that it is indeed different lifestyle behaviours and not countries heterogeneity that create this variation in the trajectories. In addition, from a methodological point of view, the fact that we considered study effect, within-country household wealth quintile, and education level in the creation of our models also enforces the belief that any heterogeneity in the study level has already been accounted for in the models specification.

These consistent findings suggest the potential for physical activity to increase baseline health and functioning and minimise decline rates in older age. Even a small amount of physical activity (such as getting involved in moderate physical activity once per week) may reduce likelihood of experiencing severe deterioration of health and functioning in older age. Among other potential pathways through which physical activity may have a beneficial effect on older adults’ health, previous evidence has suggested that active older adults, compared to inactive ones, present lower cardiometabolic risk [26, 27] and age-related inflammation [28], both of which are related to many chronic diseases.

Whilst most of the population exhibited a stable trajectory at a high level of health, we found two subgroups with alternative trajectories: one with a stable lower level of health; and a third subgroup starting at a similar point as the majority of the population but whose health severely deteriorated over time. These results were similar to those reported in previous studies in terms of the number and shape of the different trajectories [9, 29, 30], although in some of these studies the declining trajectories comprised a higher percentage of the population. Our findings were replicated in the sensitivity analyses, supporting the existence of heterogeneity in ageing trajectories and the importance of accounting for this heterogeneity when studying healthy ageing [3, 4].

### Public health implications

Empirical evidence from this study highlight the positive impact of physical activity on supporting healthy ageing, in particular the opportunity to attenuate health and functioning declines in older age. Increasing any levels of physical activity or breaking sedentary behaviour can be beneficial to health and functioning in older adults [31, 32]. Indeed, physical activity promotion has been a key focus of public health policies particularly in high income countries [33]. Several community-based and web-based interventions have been developed to promote physical activity and prevent loss of functional ability in older people [25, 34–40]. However, effectiveness of physical activity interventions might be uncertain given short follow-up periods and highly selected populations in most trials [41]. Future research may assess long-term effects of physical activity interventions and identify barriers to maintain activity levels and reduce sedentary lifestyle in older age. Utilising existing cohort data with longitudinal study designs can be a possible approach to investigate trajectories of physical activity across the life course and provide evidence to identify key factors that support physical activity habits in older adults and inform population-level interventions [42].

Public health policies on physical activity promotion can play a key role in reducing burdens of disability as well as healthcare costs in ageing populations. Although policies that promote individual-based interventions may be helpful to develop plans and approaches to increase physical activity at the individual level, it is also important to provide a supportive environment for active ageing [43] and address environmental and social factors related to physical activity in older age [44].

## Conclusions

In this study, we investigated the association between physical activity and different types of healthy ageing trajectories using a large harmonised dataset of eight cohort studies. Abstinence from any form of physical activity was associated with poor healthy ageing trajectories in terms of low baseline scores and fast decline rates. Promoting an active lifestyle appears to play an important role in maintaining health and functioning in older age.

## Supplementary information

**Additional file 1.** Additional details on methods and results.

## Data Availability

The datasets used and/or analysed during the current study are available from the corresponding author on reasonable request.

## Abbreviations

ALSA: Australian longitudinal study of ageing
ATHLOS: Ageing trajectories of health: longitudinal opportunities and synergies
BIC: Bayesian information criterion
ELSA: English longitudinal study of ageing
ENRICA: Study on cardiovascular health, nutrition and frailty in older adults in Spain
GMM: Growth mixture modelling
HRS: Health and retirement study
JSTAR: Japanese Study of ageing and retirement
KLOSA: Korean longitudinal study of ageing
LMR-LRT: Lo-Mendel-Rubin likelihood ratio test
MAR: Missing at random
MHAS: Mexican health and aging study
MLR: Maximum likelihood with errors robust to non-normality and non-independence of observations
SABIC: Sample-size adjusted bayesian information criterion
SHARE: Survey of health ageing and retirement in Europe
WHO: World health organization
