# Supplementary material for: The impact of physical activity on healthy ageing trajectories: evidence from eight cohort studies
Source: Int J Behav Nutr Phys Act. 2020 Jul 16;17:92. doi: 10.1186/s12966-020-00995-8 (PMC7364650; doi:10.1186/s12966-020-00995-8)

**Table S1.** Items used in the two-parameter logistic item response theory model.

| **Domains** | **Presence or absence of difficulties** | **ALSA** | **ELSA** | **ENRICA** | **HRS** | **JSTAR** | **KLOSA** | **MHAS** | **SHARE** |
| --- | --- | --- | --- | --- | --- | --- | --- | --- | --- |
| **Cognition** | Memory |  | x |  | x |  |  | x | x |
|  | Immediate recall * |  | x |  | x | x |  | x | x |
|  | Delayed recall * |  | x |  | x | x |  | x | x |
|  | Verbal fluency * |  | x |  |  |  |  | x | x |
|  | Orientation in time | x | x |  | x | x |  |  | x |
|  | Processing speed |  | x |  |  |  |  | x |  |
|  | Numeracy |  | x |  | x | x |  |  | x |
| **Psychological symptoms** | Sleeping | x | x | x | x | x | x | x | x |
| **Vitality** | Experiences in some degree of pain | x | x |  | x |  | x | x | x |
|  | Having high level of energy | x | x | x | x | x |  | x | x |
|  | Urine incontinence | x | x |  | x |  | x | x | x |
| **Sensory** | Near vision | x | x |  | x | x | x |  | x |
|  | Far vision | x | x |  | x | x | x |  | x |
|  | Eyesight using glasses or lens as usual | x | x |  | x | x | x | x | x |
|  | Hearing in general | x | x |  | x | x | x | x | x |
|  | Hearing in a conversation | x | x |  |  | x |  |  | x |
| **Mobility** | Stooping, kneeling or crouching | x | x | x | x | x |  | x | x |
|  | Lifting or carrying weights | x | x | x | x | x |  | x | x |
|  | Climbing stairs |  | x | x | x | x |  | x | x |
|  | Getting up from sitting down |  | x |  | x | x |  | x | x |
|  | Walking by yourself and without any equipment | x | x | x | x | x | x | x | x |
|  | Pulling or pushing large objects | x | x |  | x | x |  | x | x |
|  | Sitting for long periods | x | x |  | x | x |  | x | x |
|  | Reaching or extending arms | x | x |  | x | x |  | x | x |
|  | Walking speed * |  | x |  | x |  |  | x | x |
|  | Dizziness when walking on a level surface | x | x |  | x |  |  |  | x |
|  | Picking up things with fingers | x | x |  | x | x |  | x | x |
| **Activities of daily living** | Getting in or out of bed | x | x |  | x | x |  | x | x |
|  | Bathing or showering | x | x |  | x | x |  | x | x |
|  | Getting dressed | x | x |  |  | x |  | x | x |
|  | Moving around the home | x | x |  | x | x |  | x | x |
|  | Using the toilet | x | x |  | x | x | x | x | x |
|  | Eating | x | x |  | x | x |  | x | x |
| **Instrumental activities of daily living** | Doing housework | x | x | x | x | x | x |  | x |
|  | Shopping for groceries | x | x | x | x | x | x | x | x |
|  | Getting out of the house | x | x |  |  | x | x |  |  |
|  | Preparing meals | x | x | x | x |  | x | x | x |
|  | Using a map |  | x | x | x |  |  |  | x |
|  | Managing money, bills or expenses | x | x | x | x | x | x | x |  |
|  | Taking medications |  | x | x | x | x | x | x | x |
|  | Making telephone calls | x | x | x | x | x | x |  | x |
| *Note.* * Items dichotomised in the first quartile. | |  |  |  |  |  |  |  |  |

**Table S2.** Presence of the questions used in the aggregated variable on physical activity by study.

| Variable | ALSA | ELSA | ENRICA | HRS | JSTAR | KLOSA | MHAS | SHARE |
| --- | --- | --- | --- | --- | --- | --- | --- | --- |
| Frequency of vigorous exercise | x |  |  | x | x | x |  | x |
| Frequency of less vigorous exercise | x |  |  | x | x |  |  | x |
| Level of physical activity |  | x |  |  |  |  |  |  |
| Engagement in vigorous exercise during the last 2 weeks | x | x |  | x |  |  | x | x |
| Frequency of vigorous exercise activities in the last 2 weeks | x | x |  | x | x | x |  | x |
| Time spent doing vigorous exercise in the last 2 weeks | x |  | x |  | x | x |  |  |

**Table S3.** Guidelines for Reporting on Latent Trajectory Studies (GRoLTS) Checklist.

| **Checklist item** | **Reported?** |
| --- | --- |
| 1. Is the metric of time used in the statistical model reported? | Yes |
| 2. Is information presented about the mean and variance of time within a wave? | Yes (mean).  No (variance): not available. |
| 3a. Is the missing data mechanism reported? | Yes |
| 3b. Is a description provided of what variables are related to attrition/missing data? | Yes |
| 3c. Is a description provided of how missing data in the analyses were dealt with? | Yes |
| 4. Is information about the distribution of the observed variables included? | Yes |
| 5. Is the software mentioned? | Yes |
| 6a. Are alternative specifications of within-class heterogeneity considered (e.g., LGCA vs. LGMM) and clearly documented? If not, was sufficient justification provided as to eliminate certain specifications from consideration? | Yes |
| 6b. Are alternative specifications of the between-class differences in variance–covariance matrix structure considered and clearly documented? If not, was sufficient justification provided as to eliminate certain specifications from consideration? | Yes |
| 7. Are alternative shape/functional forms of the trajectories described? | Yes |
| 8. If covariates have been used, can analyses still be replicated? | Yes |
| 9. Is information reported about the number of random start values and final iterations included? | Yes |
| 10. Are the model comparison (and selection) tools described from a statistical perspective? | Yes |
| 11. Are the total number of fitted models reported, including a one-class solution? | Yes |
| 12. Are the number of cases per class reported for each model (absolute sample size, or proportion)? | Yes |
| 13. If classification of cases in a trajectory is the goal, is entropy reported? | Yes |
| 14a. Is a plot included with the estimated mean trajectories of the final solution? | Yes |
| 14b. Are plots included with the estimated mean trajectories for each model? | Yes |
| 14c. Is a plot included of the combination of estimated means of the final model and the observed individual trajectories split out for each latent class? | No: the vast number of individual trajectories makes such a plot unfeasible to compute and interpret. |
| 15. Are characteristics of the final class solution numerically described (i.e., means, SD/SE, n, CI, etc.)? | Yes |
| 16. Are the syntax files available (either in the appendix, supplementary materials, or from the authors)? | Yes |

*Note.* Checklist is available in Rens van de Schoot, Marit Sijbrandij, Sonja D. Winter, Sarah Depaoli & Jeroen K. Vermunt (2017) The GRoLTS-Checklist: Guidelines for Reporting on Latent Trajectory Studies, *Structural Equation Modeling: A Multidisciplinary Journal*, 24:3, 451-467, DOI: 10.1080/10705511.2016.1247646.

**Figure S1.** Linear trajectory of health from the Latent Growth Curve Model (1 class).


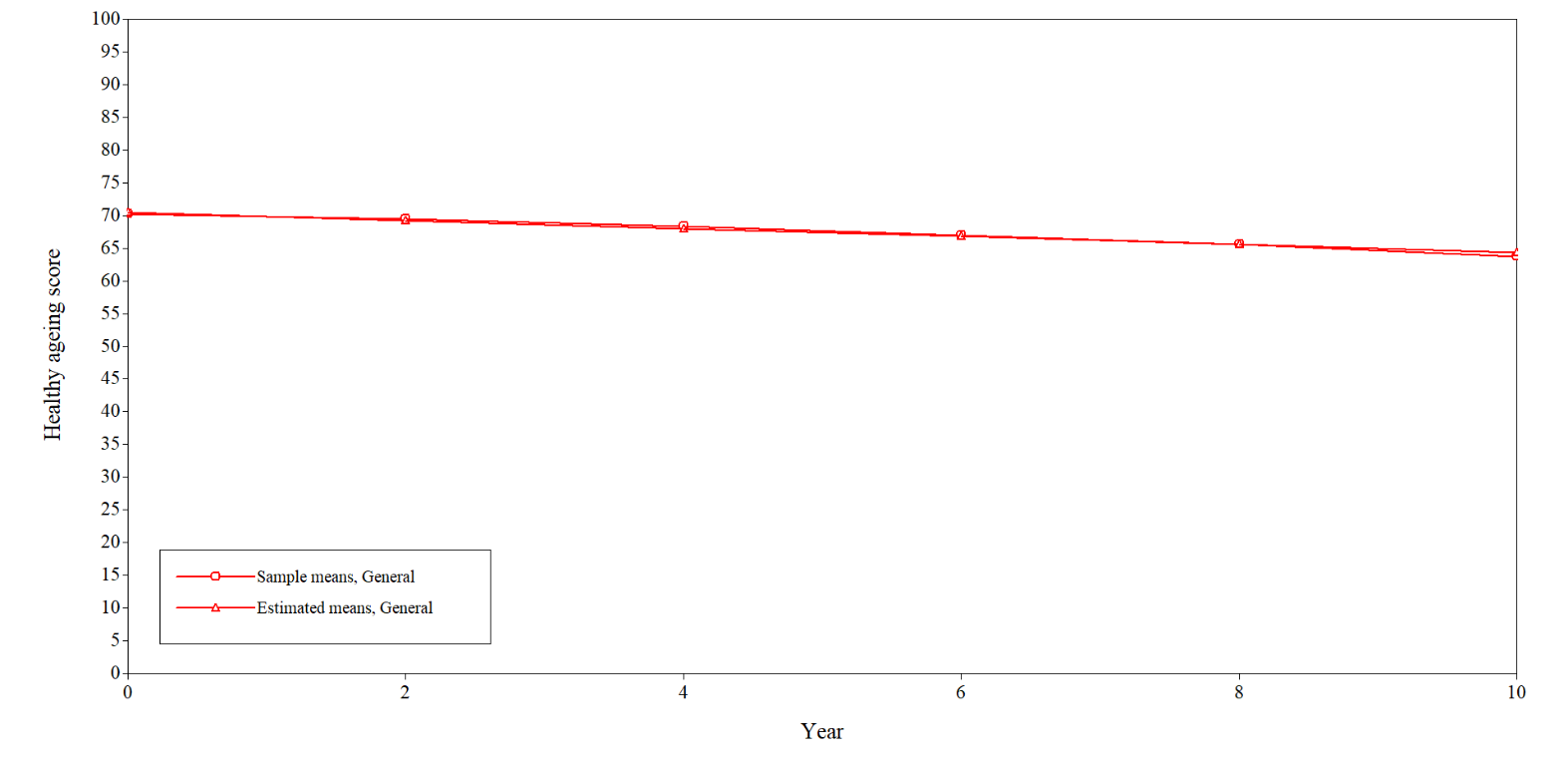


**Figure S2.** Linear trajectories of health from the Growth Mixture Model (2 classes).


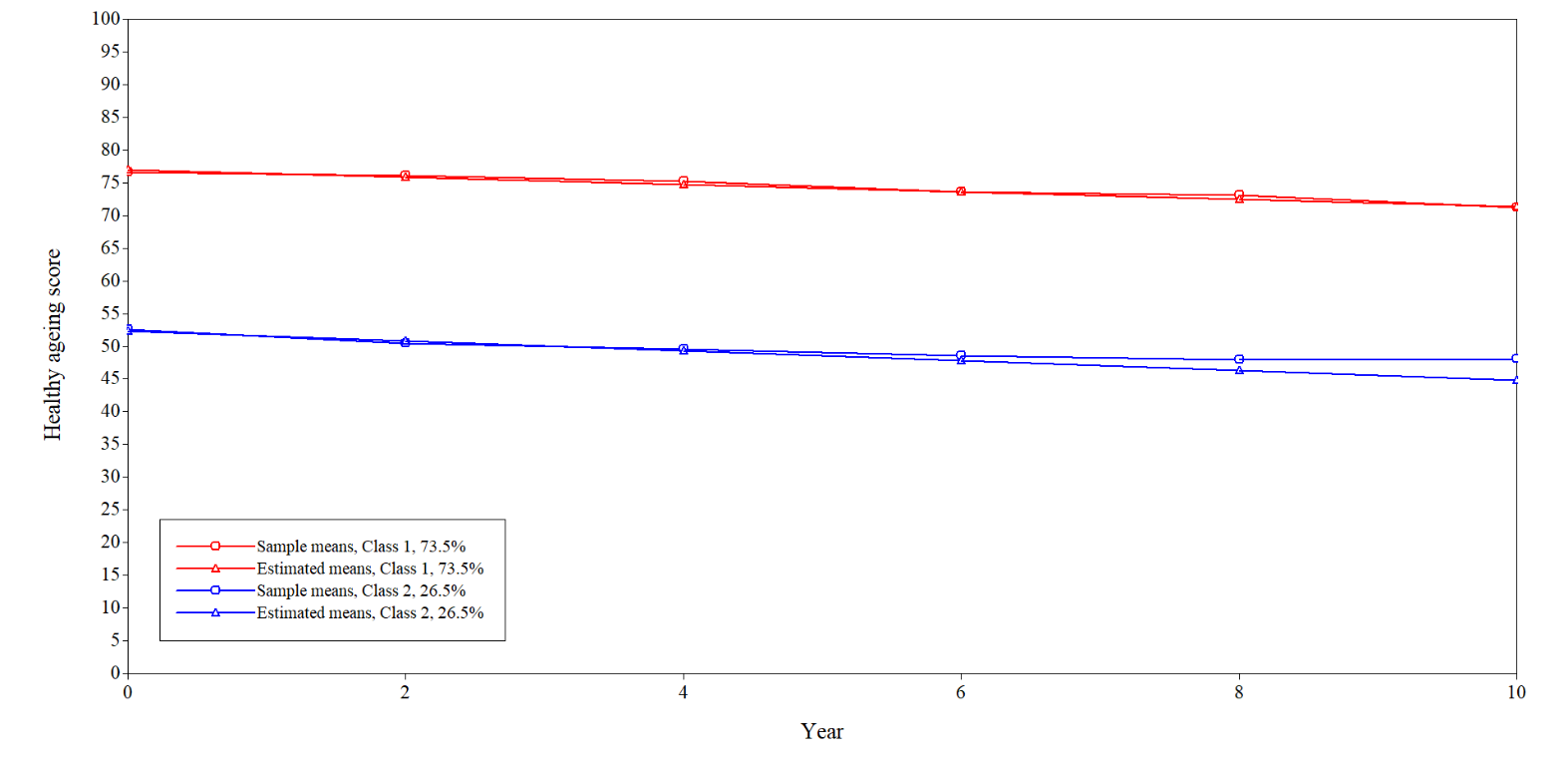


**Figure S3.** Linear trajectories of health from the Growth Mixture Model (3 classes).


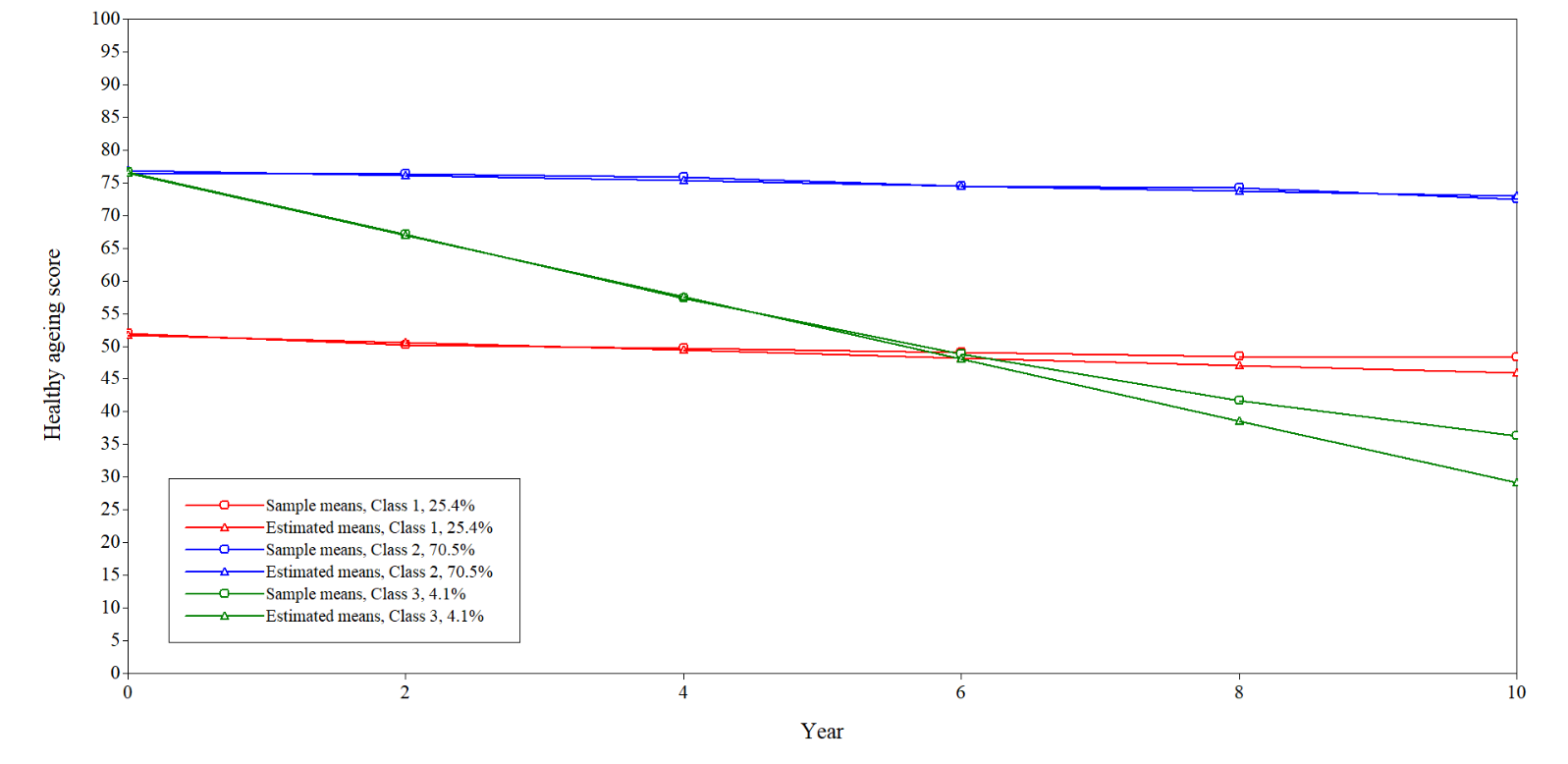


**Figure S4.** Linear trajectories of health from the Growth Mixture Model (4 classes).


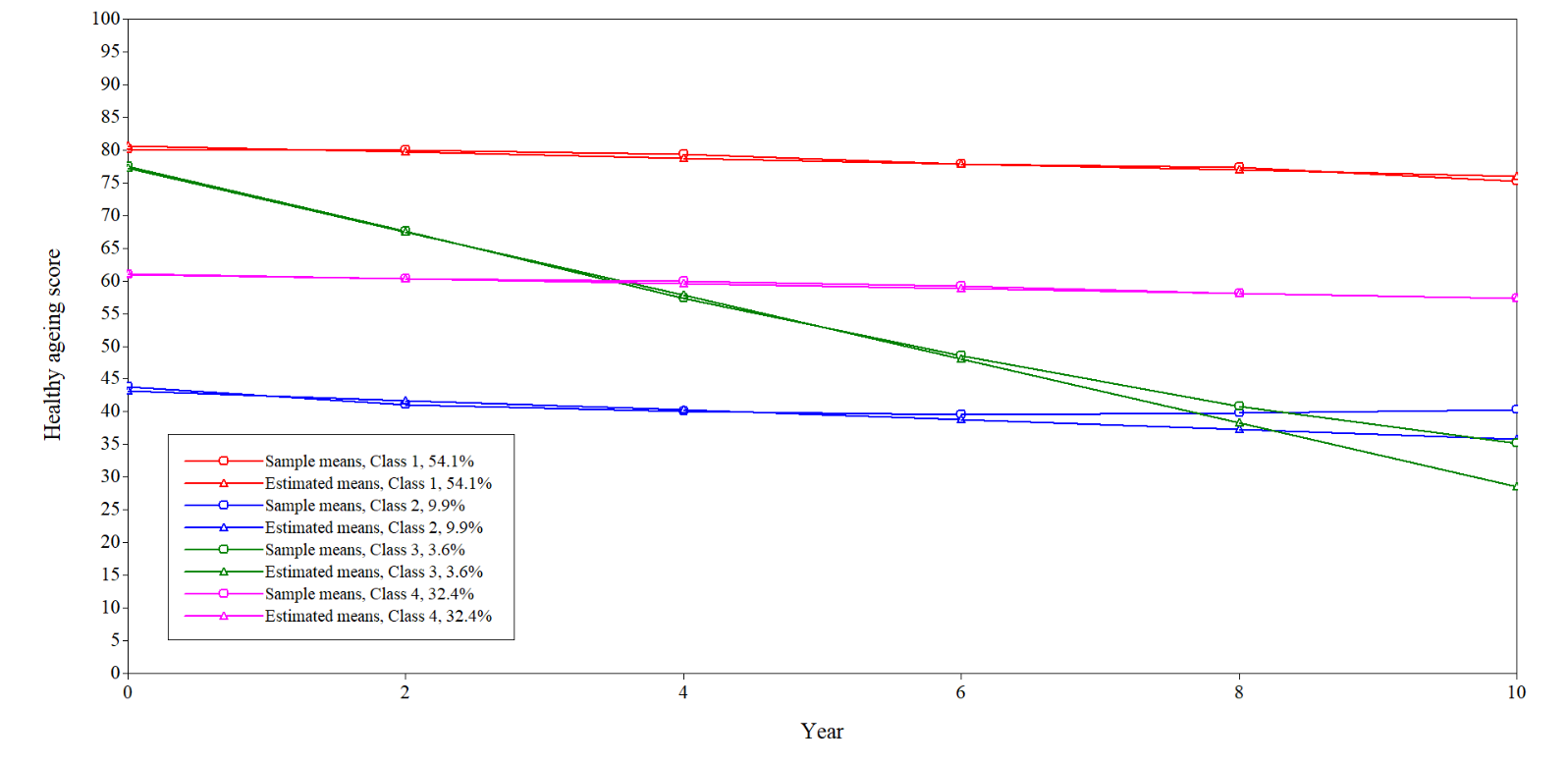


**Figure S5.** Linear trajectories of health from the Growth Mixture Model (5 classes).


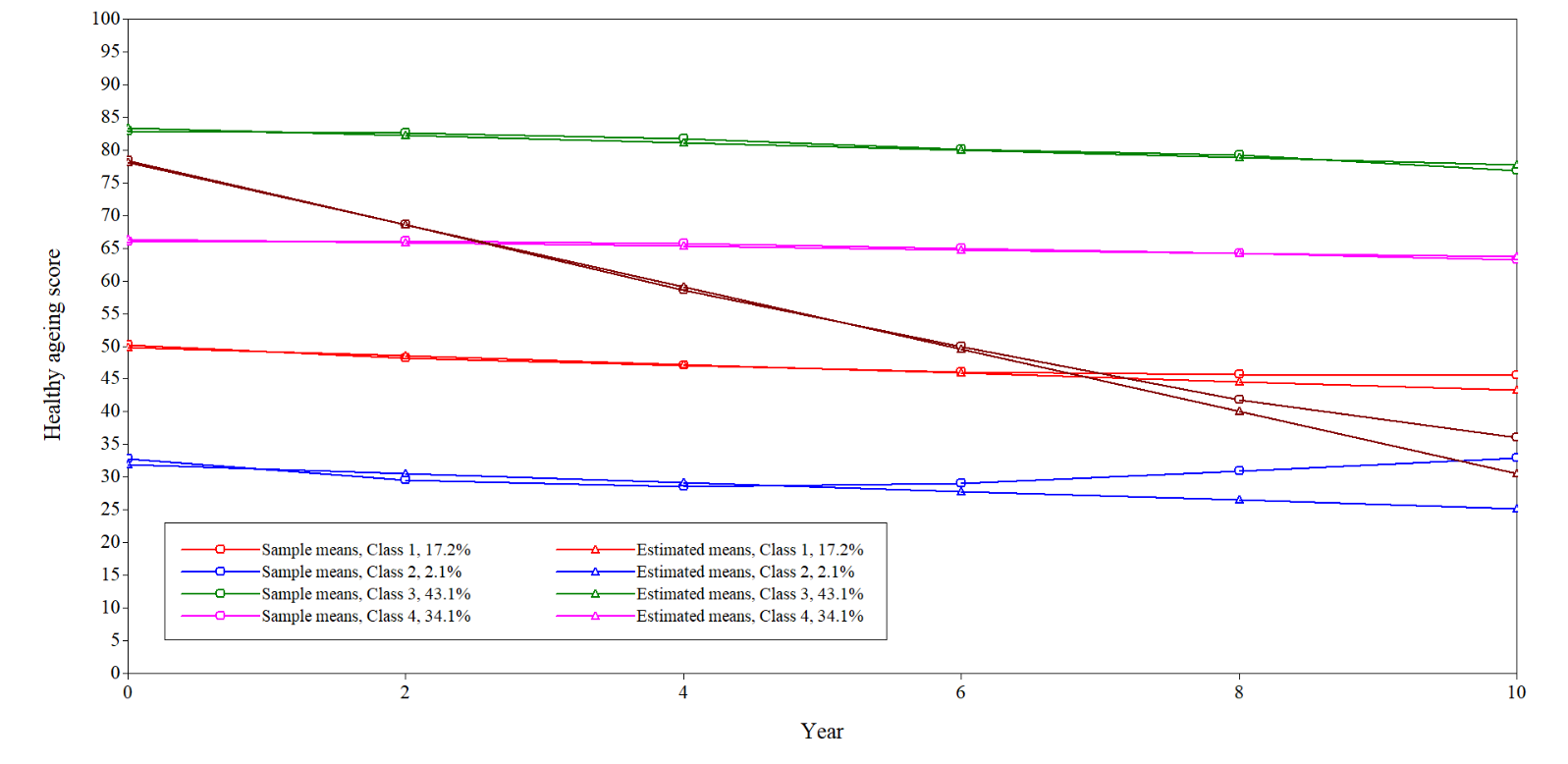


**Figure S6.** Quadratic trajectory of health from the Latent Growth Curve Model (1 class).


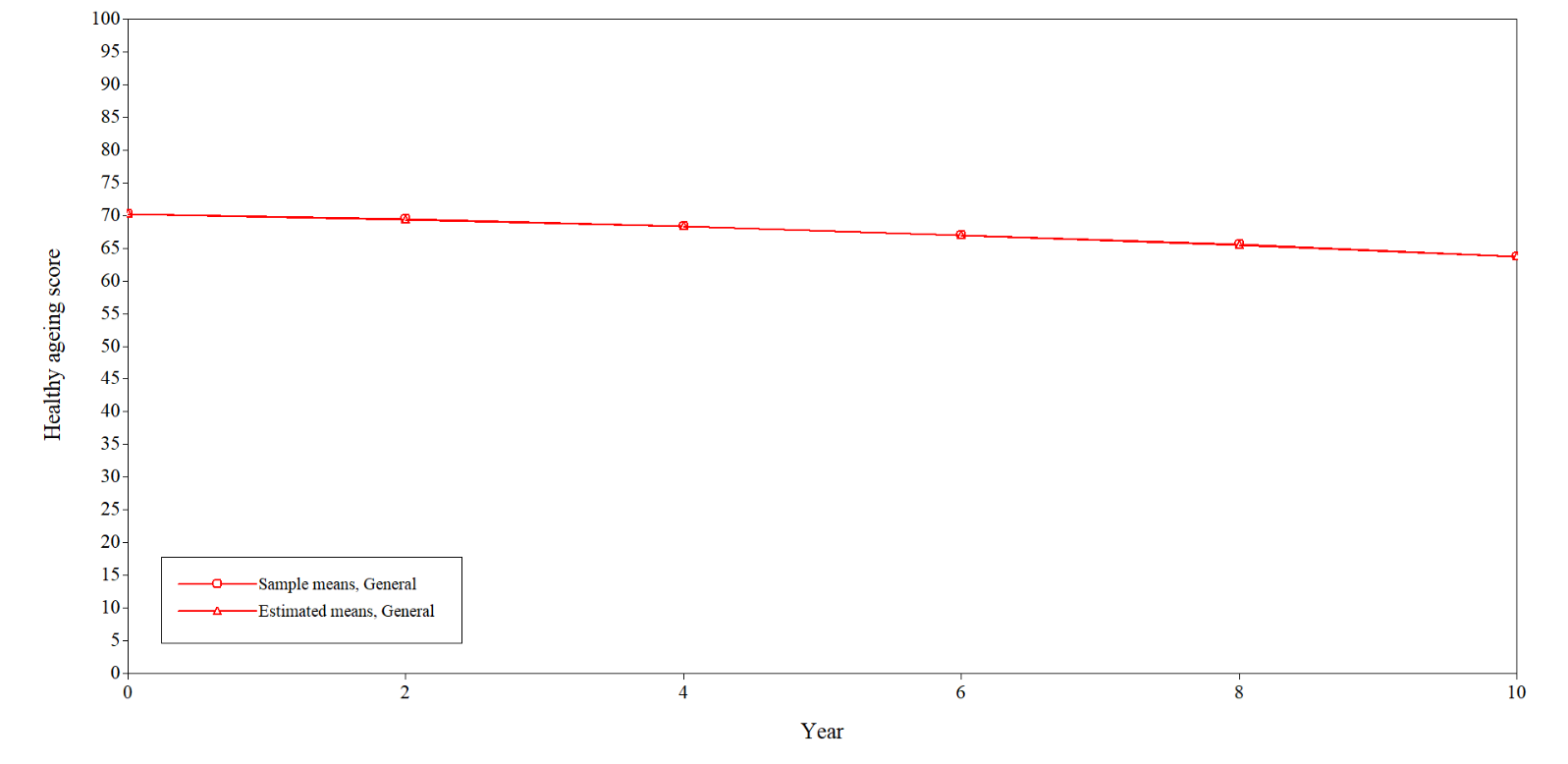


**Figure S7.** Quadratic trajectories of health from the Growth Mixture Model (2 classes).


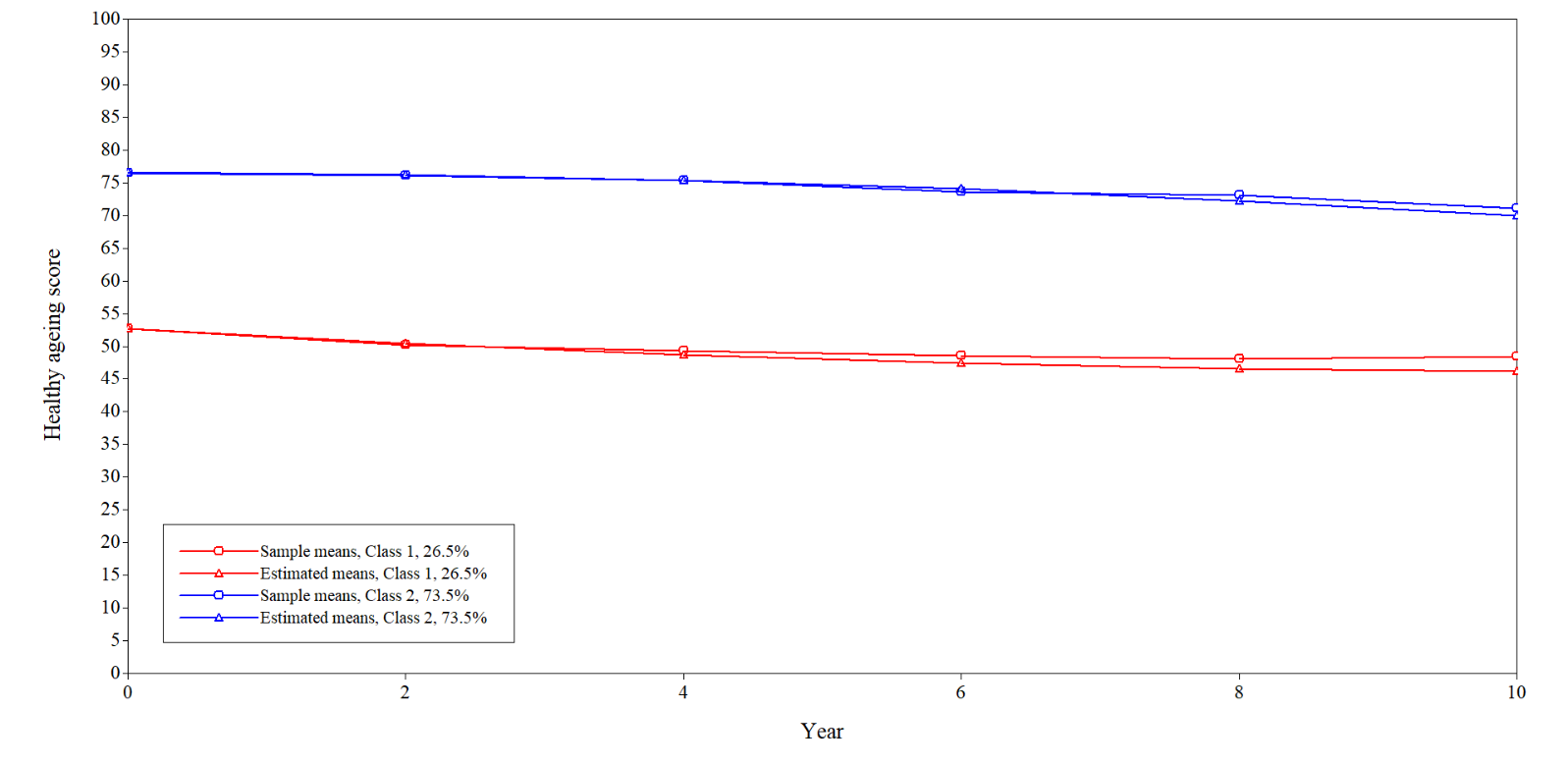


**Figure S8.** Quadratic trajectories of health from the Growth Mixture Model (3 classes).


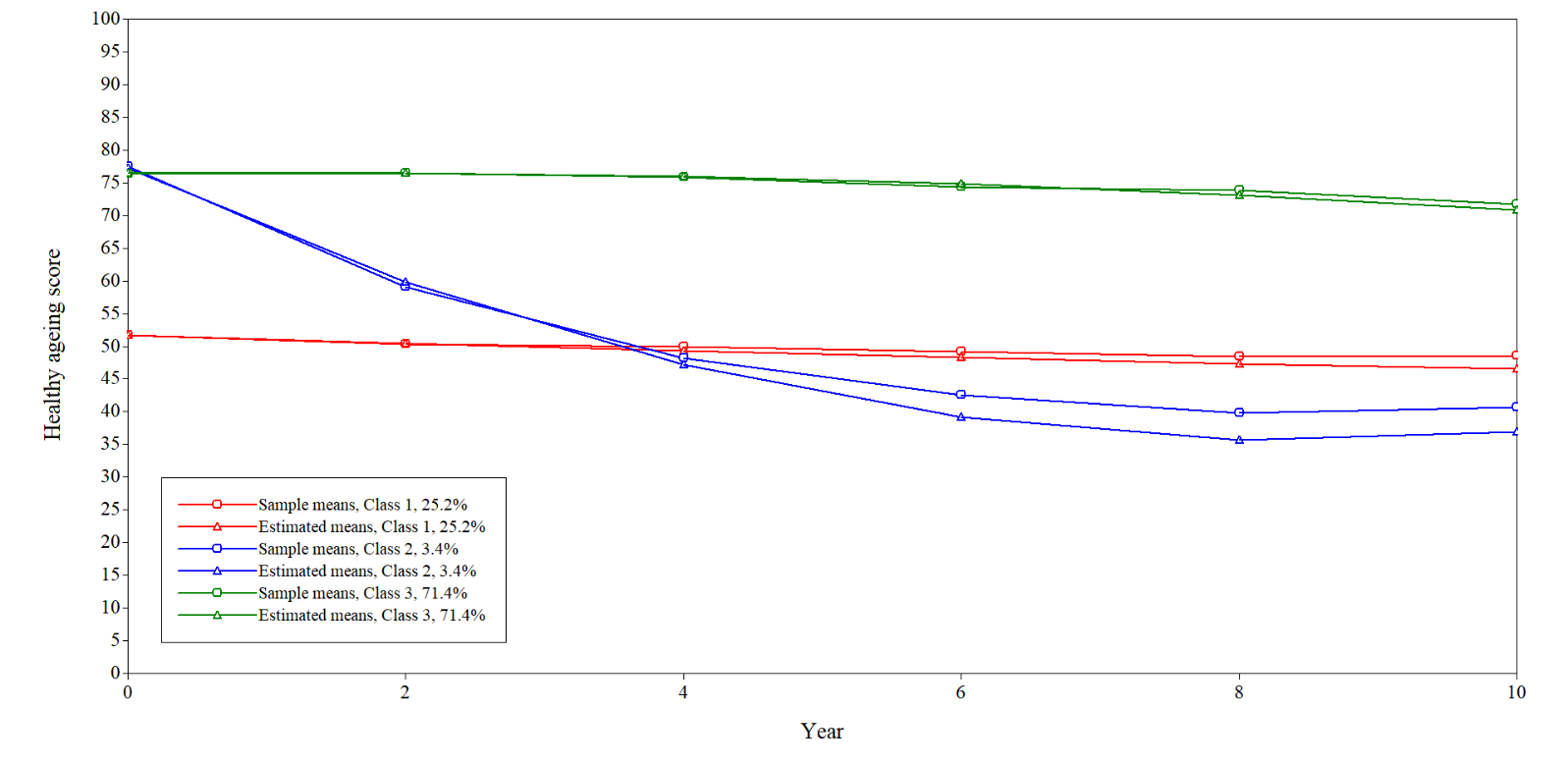


**Figure S9.** Quadratic trajectories of health from the Growth Mixture Model (4 classes).


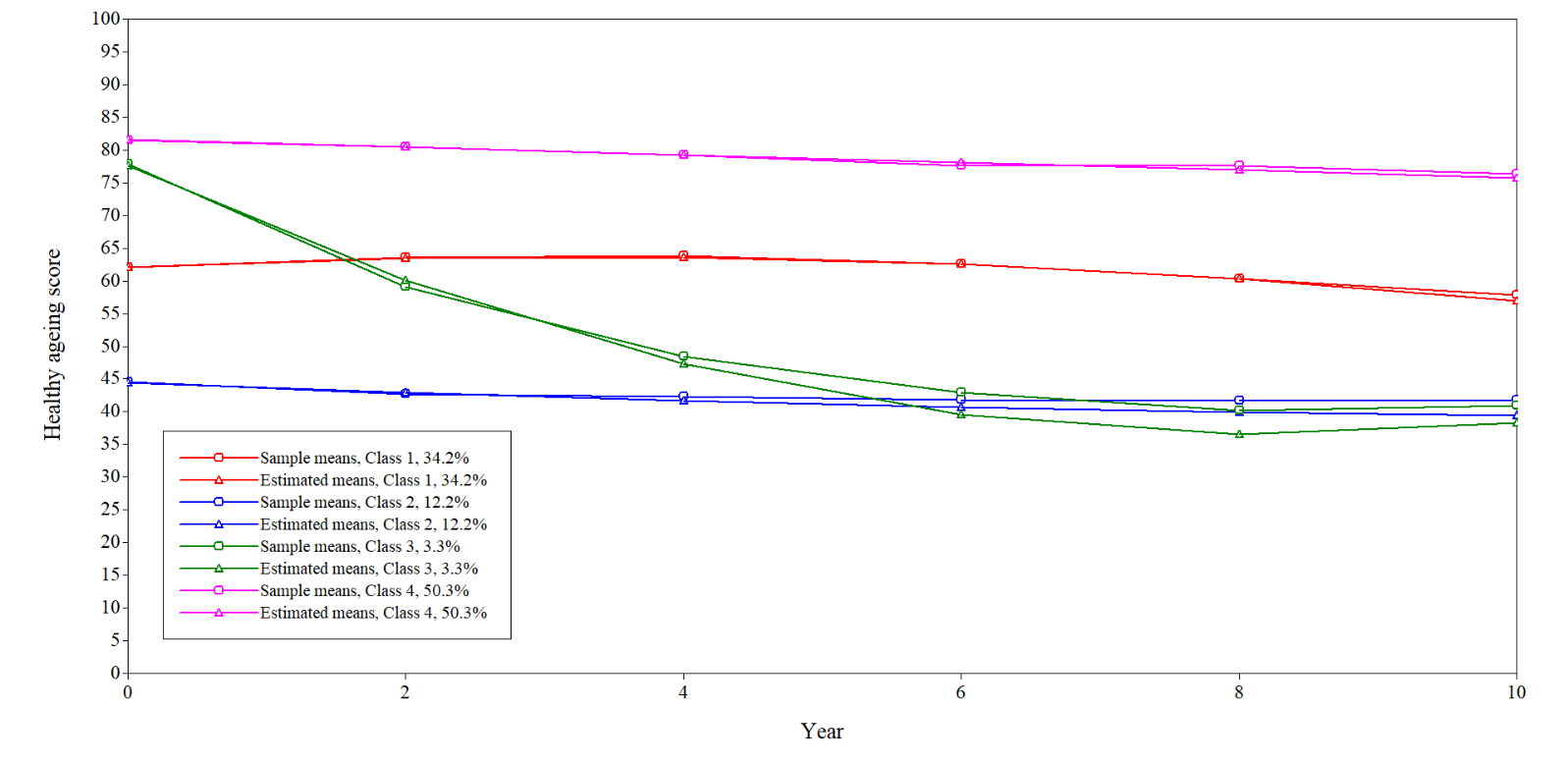

Supplement: Supplementary file 1 — Additional file 1. Additional details on methods and results. [file 12966_2020_995_MOESM1_ESM.docx]
